# Association of Job Demands with Work Engagement of Japanese Employees: Comparison of Challenges with Hindrances (J-HOPE)

**DOI:** 10.1371/journal.pone.0091583

**Published:** 2014-03-10

**Authors:** Akiomi Inoue, Norito Kawakami, Akizumi Tsutsumi, Akihito Shimazu, Koichi Miyaki, Masaya Takahashi, Sumiko Kurioka, Hisashi Eguchi, Masao Tsuchiya, Kazuhiko Enta, Yuki Kosugi, Tomoko Sakata, Takafumi Totsuzaki

**Affiliations:** 1 Department of Mental Health, Institute of Industrial Ecological Sciences, University of Occupational and Environmental Health, Japan, Kitakyushu, Japan; 2 Department of Mental Health, Graduate School of Medicine, The University of Tokyo, Tokyo, Japan; 3 Department of Public Health, Kitasato University School of Medicine, Sagamihara, Japan; 4 Division of Clinical Epidemiology, Department of Clinical Research and Informatics, National Center for Global Health and Medicine, Tokyo, Japan; 5 Health Administration and Psychosocial Factor Research Group, National Institute of Occupational Safety and Health, Japan, Kawasaki, Japan; 6 Department of Health Policy and Management, Graduate School of Medical Science, University of Occupational and Environmental Health, Japan, Kitakyushu, Japan; 7 Health Care Center, Central Japan Railway Company, Nagoya, Japan; 8 Kosugi Health Management Office, Toyama, Japan; 9 Department of Laboratory Medicine, Fukuoka Tokushukai Medical Center, Kasuga, Japan; 10 Uchisaiwaicho Medical Center, Mizuho Health Insurance Society, Tokyo, Japan; University of Utah, United States of America

## Abstract

**Objectives:**

Recent epidemiological research in Europe has reported that two groups of job demands, i.e., challenges and hindrances, are differently associated with work engagement. The purpose of the present study was to replicate the cross-sectional association of workload and time pressure (as a challenge) and role ambiguity (as a hindrance) with work engagement among Japanese employees.

**Methods:**

Between October 2010 and December 2011, a total of 9,134 employees (7,101 men and 1,673 women) from 12 companies in Japan were surveyed using a self-administered questionnaire comprising the Job Content Questionnaire, National Institute for Occupational Safety and Health Generic Job Stress Questionnaire, short 10-item version of the Effort-Reward Imbalance Questionnaire, short nine-item version of the Utrecht Work Engagement Scale, and demographic characteristics. Multilevel regression analyses with a random intercept model were conducted.

**Results:**

After adjusting for demographic characteristics, workload and time pressure showed a positive association with work engagement with a small effect size (standardized coefficient [*β*] = 0.102, Cohen’s *d* [*d*] = 0.240) while role ambiguity showed a negative association with a large effect size (*β* = −0.429, *d* = 1.011). After additionally adjusting for job resources (i.e., decision latitude, supervisor support, co-worker support, and extrinsic reward), the effect size of workload and time pressure was not attenuated (*β* = 0.093, *d* = 0.234) while that of role ambiguity was attenuated but still medium (*β* = −0.242, *d* = 0.609).

**Conclusions:**

Among Japanese employees, challenges such as having higher levels of workload and time pressure may enhance work engagement but hindrances, such as role ambiguity, may reduce it.

## Introduction

In the past decade, increased attention has been paid not only to negative aspect of work-related well-being, such as burnout and psychological distress, but also to positive aspect, such as work engagement. Work engagement is defined as “a positive, fulfilling, work-related state of mind characterized by vigor, dedication, and absorption” [1], and it can influence employee health [2].

Work engagement has frequently been studied within a theoretical framework of the job demands-resources (JD-R) model [3]. According to the JD-R model, job resources, which are defined as “physical, psychological, social, or organizational job aspects that may be functional in achieving work-related goals; reduce job demands and the associated physiological and psychological costs; and stimulate personal growth and development”, enhance work engagement [4]. More specifically, job control (or decision latitude), social support at work, extrinsic reward, feedback, and supervisory coaching are considered as job resources [5]. This theoretical assumption has been demonstrated in many cross-sectional [2], [4], [6]–[9], diary [10], [11], and prospective studies [12]–[18] conducted in Europe. In Japan, one prospective study reported that higher decision latitude was significantly associated with greater work engagement [19].

Another dimension of the JD-R model is job demands. Job demands are defined as “physical, social, or organizational job aspects that require sustained physical and/or psychological effort and are associated with certain physiological and/or psychological costs” [3]. The JD-R model theoretically does not assume any direct association of job demands with work engagement [6]. However, empirical studies based on the JD-R model have shown that some types of job demands (e.g., workload, time pressure, cognitive demands, etc.) are positively associated with work engagement both concurrently [20] and over time [16]. On the other hand, other types of job demands (e.g., role ambiguity, role conflict, etc.) are negatively associated with dedication component of work engagement over time [15], [16]. Two previous meta-analytic studies [21], [22] examined the effect of the two groups of job demands, such as “challenges” (e.g., workload, time pressure, etc.) and “hindrances” (e.g., role ambiguity, role conflict, etc.), on performance and job satisfaction. Challenges are defined as “work-related demands or circumstances that, although potentially stressful, have potential gains for individuals”. Hindrances are defined as “work-related demands or circumstances that tend to constrain or interfere with an individual’s work achievement” [23]. These meta-analytic studies showed a positive effect of challenges and a negative effect of hindrances on performance and job satisfaction. A recent study on work engagement conducted in Belgium and the Netherlands [24] observed similar patterns. In Japan, one prospective study reported that higher workload and time pressure, which can be considered as a challenge, were positively associated with greater work engagement [19]. However, the association of hindrances with work engagement has not yet been fully investigated.

The purpose of the present study was to investigate a cross-sectional association of two groups of job demands, i.e., challenges and hindrances, with work engagement among Japanese employees. It was hypothesized that challenges would be positively and hindrances would be negatively associated with work engagement. Van den Broeck et al. [24] claimed that considering job resources in conjunction with these two groups of job demands might further add to the comprehensiveness of the JD-R model and enrich its theoretical and practical value. Therefore, the present study included job resources based on the job demands-control (JD-C) (or demand-control-support [DCS]) and effort-reward imbalance (ERI) models [25]–[27] (i.e., decision latitude, supervisor support, co-worker support, and extrinsic reward) in the analyses. Furthermore, because a previous study reported that work engagement is particularly enhanced when both job demands and job resources are high [6], the present study also included interaction terms of each job demands component with job resources component in the model.

## Methods

### Participants

We used a cross-sectional data from the baseline survey of an occupational cohort study on social class and health conducted in Japan (Japanese Study of Health, Occupation, and Psychosocial Factors Related Equity: J-HOPE), which was conducted between October 2010 and December 2011. The study population consisted of employees representing a number of different industries and a wide variety of occupations. The primary industry sectors represented were hospitals and medical facilities; transportation (a railway company); manufacturing; and the information technology, pharmaceutical, and service industries. The original sample comprised 10,807 employees from 12 companies (response rate = 77.4%). The participants were surveyed using a self-administered questionnaire measuring job demands, job resources, work engagement, and demographic characteristics. After excluding 1,673 employees who had at least one missing response for variables relevant to this study, the data from 9,134 employees (7,101 men and 1,673 women) were analyzed. Compared to the excluded sample (*n* = 1,673), the final sample (*n* = 9,134) was significantly younger, and it comprised a significantly higher percentage of professionals, technicians, labourers, and individuals with higher education. The two groups did not differ in other demographic characteristics (i.e., gender, number of family members, or shift work). Detailed characteristics of participants are shown in Table 1.

**Table 1:** Demographic characteristics, job demands, job resources, and work engagement among employees who participated in the study (*N* = 9,134).

Demographic characteristics	Average (SD)†	*n* (%)
Gender		
Men		7,101 (77.7)
Women		2,033 (22.3)
Age (years)	40.6 (10.5)	
Education (years)	14.3 (2.60)	
More than 12 years		5,656 (61.9)
12 years or less		3,478 (38.1)
Number of family members	2.95 (1.52)	
Occupation		
Managers		1,633 (17.9)
Professionals		1,303 (14.3)
Technicians		1,018 (11.1)
Clerks		1,303 (14.3)
Service and sales workers		496 (5.4)
Craft and related trades workers		567 (6.2)
Machine operators and assemblers		982 (10.8)
Laborers		766 (8.4)
Others		1,066 (11.7)
Shift work		
Day shift only		7,439 (81.4)
Shift work with night duty		1,354 (14.8)
Shift work without night duty		212 (2.3)
Night shift only		129 (1.4)
**Scale scores** ‡	**Average (SD)** †	**Cronbach’s α**
Job demands (challenge)		
Workload and time pressure (JCQ)	32.8 (5.45)	0.69
Job demands (hindrance)		
Role ambiguity (NIOSH-GJSQ)	17.9 (5.85)	0.87
Job resources		
Decision latitude (JCQ)	66.5 (10.2)	0.78
Supervisor support (JCQ)	11.1 (2.35)	0.90
Co-worker support (JCQ)	11.4 (1.84)	0.80
Extrinsic reward (ERIQ)	18.2 (2.97)	0.74
Work engagement (UWES-9)	2.92 (0.95)	0.93

† Standard deviation.

‡ JCQ: Job Content Questionnaire; NIOSH-GJSQ: National Institute for Occupational Safety and Health Generic Job Stress Questionnaire; ERIQ: Effort-Reward Imbalance Questionnaire; UWES: Utrecht Work Engagement Scale.

Study purposes and procedures were explained and written informed consent was obtained from the employees prior to the initiation of the study. The Ethics Committee of the Graduate School of Medicine/Faculty of Medicine, The University of Tokyo, Kitasato University School of Medicine/Hospital, and University of Occupational and Environmental Health, Japan reviewed and approved the aims and procedures of this study (No. 2772, B12-103, and 10-004, respectively).

### Measures

#### 1) Job demands

In the present study, according to previous studies in Europe [21]–[23], we used workload and time pressure as a challenge and role ambiguity as a hindrance. Workload and time pressure were assessed using the Japanese version of the Job Content Questionnaire (JCQ) [28], [29]. The JCQ has a five-item scale measuring workload and time pressure (response range = 12–48). The Japanese version of the JCQ was reported to have acceptable internal consistency reliability and construct validity [29]. In this sample, Cronbach’s alpha coefficient was 0.69 (Table 1). The total score was calculated according to the JCQ User’s Guide [28]. On the other hand, role ambiguity was assessed using the Japanese version of the National Institute for Occupational Safety and Health Generic Job Stress Questionnaire (NIOSH-GJSQ) [30], [31], which includes a six-item role ambiguity scale (response range = 6–42). The Japanese version of the NIOSH-GJSQ was also reported to have acceptable internal consistency reliability and construct validity [31]. In this sample, Cronbach’s alpha coefficient was 0.87. Each scale score was used as a continuous variable.

#### 2) Job resources

Concerning job resources, we measured decision latitude, supervisor support, co-worker support, and extrinsic reward, based on the JD-C (or DCS) and ERI models. Decision latitude, supervisor support, and co-worker support were also assessed using the Japanese version of the JCQ [28], [29], which includes a nine-item decision latitude scale (response range = 24–96), a four-item supervisor support scale (response range = 4–16), and a four-item co-worker support scale (response range = 4–16). In this sample, Cronbach’s alpha coefficients were 0.78, 0.90, and 0.80 for decision latitude, supervisor support, and co-worker support, respectively. On the other hand, extrinsic reward was assessed using the 10-item Japanese short version of the Effort-Reward Imbalance Questionnaire (ERIQ) [32], [33], which includes a seven-item extrinsic reward scale (response range = 7–28). The 10-item Japanese short version of the ERIQ was reported to have acceptable internal consistency reliability and construct validity [33]. In this sample, Cronbach’s alpha coefficient was 0.74. Each scale score was used as a continuous variable.

#### 3) Work engagement

The nine-item Japanese version of the Utrecht Work Engagement Scale (UWES-9) [34], [35] was used to assess work engagement. The UWES-9, developed by Schaufeli et al. [34], includes scales measuring vigor (three items), dedication (three items), and absorption (three items) on a seven-point scale ranging from 0 = *never* to 6 = *always (everyday)*. The UWES-9 was translated into Japanese, and the internal consistency reliability and construct validity are acceptable for this version [35]. In this sample, Cronbach’s alpha coefficient was 0.93. According to the UWES test manual [36], a total score for the UWES-9 (range 0–6) was calculated by averaging items and was used as a continuous variable. Average scale scores and Pearson’s correlation coefficients among job demands, job resources, and work engagement are shown in Tables 1 and 2, respectively.

**Table 2:** Pearson’s correlation coefficients for job demands, job resources, and work engagement (7,101 men and 2,033 women)†.

Variables	1	2	3	4	5	6	7
1. Workload and time pressure		0.059	0.220	−0.052	0.046	−0.097	0.097
2. Role ambiguity	0.047		−0.345	−0.420	−0.340	−0.429	−0.449
3. Decision latitude	0.237	−0.357		0.309	0.305	0.292	0.398
4. Supervisor support	−0.047	−0.422	0.310		0.465	0.469	0.315
5. Co-worker support	0.045	−0.338	0.295	0.465		0.392	0.308
6. Extrinsic reward	−0.085	−0.435	0.306	0.470	0.389		0.347
7. Work engagement	0.105	−0.454	0.405	0.317	0.306	0.352	

† All coefficients were significant at the *p*<0.01 level. Crude correlations are on the lower diagonal, and partial correlations adjusted for age are on the upper diagonal.

#### 4) Demographic characteristics

Demographic characteristics included gender, age, education, number of family members, occupation, and shift work, which were used as covariates in previous studies [16]. Age and number of family members were used as continuous variables. Education was dichotomized into some college or higher (i.e., more than 12 years) and senior high school or less (i.e., 12 years or less). Occupation was classified into five non-manual worker groups (i.e., managers, professionals, technicians, clerks, and service and sales workers) and four manual worker groups (i.e., craft and related trade workers, machine operators and assemblers, laborers, and others) using the original classification, and dummy variables were created using managers as a reference. Shift work was classified into four groups using the original classification (i.e., day shift only, shift work with night duty, shift work without night duty, and night shift only), and dummy variables were created using the day shift only group as a reference.

### Statistical Analysis

Since our data had a multilevel structure, comprising employees nested within companies, we conducted multilevel regression analyses with a random intercept model. The model included individual-level factors: demographic characteristics (i.e., gender, age, education, number of family members, occupation [dummy variables with the managers as a reference], and shift work [dummy variables with the day shift only group as a reference]), job demands (i.e., workload and time pressure and role ambiguity), and job resources (i.e., decision latitude, supervisor support, co-worker support, and extrinsic reward). The model applied the centring approach in which individual-level of job demands and job resources were centred by subtracting the group means. When we calculated the intraclass correlation coefficients (ICCs) for each job demands and job resources component, all the ICCs were less than 0.10, which means the most variance was situated the individual-level. Therefore, company-level factors were not included in the model. Taking work engagement as a dependent variable, the analyses were expanded from a model, which included only demographic characteristics, to a model that included job demands and job resources. More specifically, demographic characteristics were entered in the first step of the analyses (Model 1) since these variables were used as covariates in previous studies [16]. Second, to examine the association of job demands with work engagement adjusted for demographic characteristics, workload and time pressure and role ambiguity were additionally entered in the model (Model 2). Third, to examine the unique association of job demands with work engagement independent of job resources, decision latitude, supervisor support, co-worker support, and extrinsic reward were additionally entered in the model (Model 3). Finally, to determine whether the association of job demands with work engagement differed by the levels of job resources, interaction terms of each job demands component with job resources component were additionally included in the model (Model 4). In addition, to examine gender (men vs. women) and occupational (non-manual workers vs. manual workers) differences in the association of job demands and job resources with work engagement, we tested the gender-interaction and occupational-interaction effects. In a series of analyses, standardized fixed effect coefficients (*β*), variance of individual-level random effect (i.e., residual) (*σ_e_^2^*), variance of company-level random effect (*σ_u_^2^*), and their 95% confidence intervals (CIs) were calculated in each model. However, when we calculated required sample size for regression analysis considering R^2^ = 0.15 [15], [16], [19], number of predictors = 21 (including dummy variables), statistical power level = 0.80, and probability level = 0.05 by G*Power 3.1 [37], we obtained minimum required sample size of 139. Therefore, the present sample size (*n* = 9,134) is unnecessarily large and can lead to “statistically” significant results even if they are not “practically” significant. We thus calculated the effect sizes (*d* = |2*β*/*σ_e_*|) [38] of each job demands and job resources component rather than using *p* levels of 0.05. According to Cohen’s suggestion [39], we interpreted *d* values of 0.2<*d*<0.5, 0.5<*d*<0.8, and 0.8<*d* as small, medium, and large effect sizes, respectively. The statistical analyses were conducted using IBM SPSS Statistics 19 for Windows.

## Results

Table 3 shows the results of multilevel regression analyses (i.e., standardized fixed effect coefficients [*β*], variance of individual-level random effect [*σ_e_^2^*], variance of company-level random effect [*σ_u_^2^*], and their 95% CIs for each model). After adjusting for demographic characteristics (Model 2), the result indicated a small positive association of workload and time pressure with work engagement (*β* = 0.102, *d* = 0.240) and a large negative association of role ambiguity with work engagement (*β* = −0.429, *d* = 1.011). After additionally adjusting for job resources (i.e., decision latitude, supervisor support, co-worker support, and extrinsic reward) (Model 3), almost the same effect size of workload and time pressure on work engagement as in Model 2 was observed (*β* = 0.093, *d* = 0.234) while the effect size of role ambiguity on work engagement was attenuated but still medium (*β* = −0.242, *d* = 0.609). After adjusting for demographic characteristics and job demands (i.e., workload and time pressure and role ambiguity) (Model 3), a medium positive association of decision latitude (*β* = 0.201, *d* = 0.507) and small positive associations of co-worker support (*β* = 0.094, *d* = 0.235) and extrinsic reward (*β* = 0.176, *d* = 0.443) with work engagement were observed. However, the effect size of supervisor support on work engagement was quite small (*β* = 0.044, *d* = 0.111). When we additionally included interaction terms of each job demands component with job resources component (Model 4), all interaction effects on work engagement were quite small (*β* = −0.038 to 0.022, *d* = 0.014 to 0.096). Furthermore, when we tested the gender-interaction effects, quite small gender-interaction effects were observed (*β* = −0.034 to 0.051, *d* = 0.008 to 0.127). In a similar way, quite small occupational-interaction effects were observed (*β* = −0.025 to 0.032, *d* = 0.002 to 0.081) (data available upon request).

**Table 3:** Association of demographic characteristics, job demands, and job resources with work engagement: multilevel analysis with random intercept model (7,101 men and 2,033 women)†.

Variables	Estimates (95% confidence interval)			
	Model 1	Model 2	Model 3	Model 4
**Fixed effect** (standardized coefficients: *β*)				
**Demographic characteristics**				
Gender†	−0.086 (−0.108, −0.063)	−0.058 (−0.078, −0.037)	−0.012 (−0.032, 0.007)	−0.011 (−0.031, 0.008)
Age	0.071 (0.049, 0.095)	0.079 (0.058, 0.100)	0.178 (0.158, 0.198)	0.177 (0.157, 0.198)
Education‡	0.054 (0.030, 0.078)	0.043 (0.021, 0.064)	0.026 (0.006, 0.046)	0.026 (0.006, 0.046)
Number of family members	0.043 (0.022, 0.064)	0.027 (0.008, 0.046)	0.024 (0.006, 0.041)	0.023 (0.006, 0.040)
Occupation (reference = managers)				
Professionals	−0.077 (−0.104, −0.050)	−0.014 (−0.039, 0.011)	0.001 (−0.022, 0.024)	0.005 (−0.018, 0.028)
Technicians	−0.114 (−0.139, −0.089)	−0.042 (−0.065, −0.020)	−0.024 (−0.045, −0.003)	−0.020 (−0.041, 0.001)
Clerks	−0.137 (−0.163, −0.110)	−0.047 (−0.072, −0.023)	0.003 (−0.020, 0.026)	0.006 (−0.017, 0.029)
Service and sales workers	−0.061 (−0.084, −0.037)	−0.017 (−0.038, 0.004)	0.016 (−0.004, 0.036)	0.018 (−0.001, 0.038)
Craft and related trades workers	−0.090 (−0.113, −0.067)	−0.031 (−0.052, −0.010)	0.000 (−0.019, 0.020)	0.002 (−0.017, 0.022)
Machine operators and assemblers	−0.149 (−0.178, −0.120)	−0.063 (−0.089, −0.037)	0.002 (−0.023, 0.026)	0.005 (−.020, 0.029)
Laborers	−0.121 (−0.146, −0.095)	−0.045 (−0.068, −0.022)	0.023 (0.000, 0.045)	0.023 (0.003, 0.047)
Others	−0.115 (−0.140, −0.089)	−0.038 (−0.061, −0.015)	0.012 (−0.010, 0.034)	0.015 (−0.007, 0.037)
Shift work (reference = day shift only)				
Shift work with night duty	−0.048 (−0.073, −0.022)	−0.037 (−0.060, −0.015)	0.005 (−0.016, 0.026)	0.007 (−0.014, 0.029)
Shift work without night duty	−0.026 (−0.046, −0.005)	−0.012 (−0.030, 0.006)	−0.004 (−0.021, 0.013)	−0.005 (−0.022, 0.012)
Night shift only	−0.028 (−0.048, −0.008)	−0.028 (−0.046, −0.010)	0.001 (−0.015, 0.018)	0.001 (−0.016, 0.018)
**Job demands** §				
Workload and time pressure		0.102 (0.084, 0.120)	0.093 (0.075, 0.111)	0.096 (0.078, 0.114)
Role ambiguity		−0.429 (−0.447, −0.411)	−0.242 (−0.262, −.222)	−0.251 (−0.271, −0.231)
**Job resources** §				
Decision latitude			0.201 (0.181, 0.222)	0.199 (0.178, 0.219)
Supervisor support			0.044 (0.024, 0.064)	0.044 (0.023, 0.065)
Co-worker support			0.094 (0.074, 0.113)	0.098 (0.078, 0.117)
Extrinsic reward			0.176 (0.155, 0.197)	0.172 (0.152, 0.193)
**Interaction (job demands × job resources)**				
Workload and time pressure × decision latitude				0.013 (−0.004, 0.031)
Workload and time pressure × supervisor support				0.011 (−0.009, 0.031)
Workload and time pressure × co-worker support				−0.022 (−0.041, −0.003)
Workload and time pressure × extrinsic reward				0.022 (0.002, 0.042)
Role ambiguity × decision latitude				−0.007 (−0.026, 0.013)
Role ambiguity × supervisor support				−0.006 (−0.029, 0.018)
Role ambiguity × co-worker support				0.003 (−0.018, 0.024)
Role ambiguity × extrinsic reward				−0.038 (−0.060, −0.016)
**Random effect** (variance)				
Individual (residual) (*σ_e_^2^*)	0.899 (0.874, 0.926)	0.720 (0.699, 0.741)	0.632 (0.614, 0.651)	0.630 (0.612, 0.649)
Company (*σ_u_^2^*)	0.057 (0.023, 0.142)	0.057 (0.023, 0.140)	0.062 (0.025, 0.150)	0.062 (0.025, 0.153)
Akaike Information Criterion (AIC)	25083.447	23071.299	21920.016	21941.845

† Men = 0, women = 1.

‡ 12 years or less = 0, more than 12 years = 1.

§ Each component of job demands and job resources was group-mean centered.

## Discussion

The present study showed a small positive association of workload and time pressure and a large negative association of role ambiguity with work engagement after adjusting for demographic characteristics. After additionally adjusting for job resources based on the JD-C (or DCS) and ERI models, the effect size of workload and time pressure was not attenuated while that of role ambiguity was attenuated but still medium. The effect sizes of interaction terms of each job demands component with job resources component on work engagement were quite small. These results were similar for men and women as well as for non-manual workers and manual workers.

Workload and time pressure were positively associated with work engagement. This is consistent with the finding from a cross-sectional study conducted in the Netherlands [20] and findings from the prospective studies conducted in Finland and Japan [16], [19], which showed positive associations of workload, cognitive demands, and time pressure with work engagement and its components (i.e., vigor, dedication, and absorption). The present findings are also consistent with those from meta-analytic studies that showed a positive effect of challenge components of job demands on performance and job satisfaction [21], [22]. The present study supports our hypothesized association of challenge components of job demands with work engagement among Japanese employees, as observed in European countries. Although it is not clear from the present study why and how workload and time pressure enhance work engagement, some unexamined factors might explain this phenomenon. For example, feelings of self-respect and importance, such as organization-based self-esteem (OBSE), could mediate the association of workload and time pressure with work engagement [40]. Being busy at work may create a feeling of being important for their company or organization, which may in turn enhance work engagement. Because Japanese society tends to expect employees to work hard [41], this pattern may be stronger for Japanese employees. Further study should try to identify factors that might mediate the association of workload and time pressure or challenge components of job demands with work engagement.

Role ambiguity was negatively associated with work engagement. This finding is also consistent with a prospective study conducted in Spain [15], which showed negative association of role ambiguity with dedication component of work engagement, as well as with meta-analytic studies, which showed a negative effect of hindrance components of job demands on performance and job satisfaction [21], [22]. The present study supports our hypothesized association of hindrance components of job demands with work engagement among Japanese employees. If employees cannot understand their own performance expectations, they cannot feel responsible for or committed to their work [42], which may in turn reduce work engagement. From this perspective, the present finding may suggest a positive association of role clarity with work engagement. In that sense, work engagement could be enhanced if employees understood their own performance expectations or responsibility. Perhaps, it might be more difficult for workers to cope with role ambiguity than with workload and time pressure, since higher authority is required to control role ambiguity [43]. Further study on more detailed mechanism underlying the association of role ambiguity (or role clarity) with work engagement is needed.

The present study found quite small effect sizes for interaction terms of each job demands component with job resources component in terms of predicting work engagement. Decision latitude, supervisor support, and co-worker support interacted more with workload and time pressure slightly than with role ambiguity, while extrinsic reward did more with role ambiguity than with workload and time pressure. However, differences in these effect sizes were again negligible. A previous study in Finland [6] showed a greater interaction effect of high job demands with high job resources on work engagement than those observed in the present study. However, this previous study was conducted with school teachers and measured pupil misbehavior as a type of job demands. The differences in the study population and measures may explain the discrepancy between the present study and previous one regarding the interaction effect of job demands with job resources. A possible reason of quite small and non-specific interaction effects of job demands with job resources is that job demands and job resources measured using the JCQ and ERIQ in the present study are broad, while these are recognized as important domains of psychosocial factors at work. According to the matching hypothesis [44], a job resource would have a greater modifying effect on a job demand especially when both are specifically matched. For example, in a previous study, an effect of workload was more effectively buffered by performance feedback than by decision latitude or social support at work [45]. On the other hand, a negative effect of role ambiguity on work engagement was buffered by leadership quality because better quality of leadership provides employees with clearer job responsibility [43]. Interaction effects of challenge and hindrance with job resources on work engagement may be greater depending on a combination of these specific components. To understand the interaction effect of job demands with job resources further, a future study should focus on more specific components of job demands and job resources, as well as their theoretical match.

In the present study, decision latitude, co-worker support, and extrinsic reward were positively associated with work engagement. This finding is consistent with the original JD-R model [4] as well as with so many empirical studies [2], [6]–[19]. On the other hand, the effect size of supervisor support on work engagement was quite small. This finding is not consistent with previous studies conducted in European countries [6]–[18]. However, a previous prospective study in Japan showed insignificant association of supervisor support with work engagement [19]. Because Asian countries, including Japan, have distinct features of workplace culture in that they tend to be more vertical and hierarchy-oriented compared to European countries [46], subordinates who obtain greater support from their supervisors may feel sorry to trouble, which may lead to weaker association of supervisor support with work engagement. To provide more concrete evidence for the association of job resources with work engagement in Japan, future research should consider culture differences as well as other kind of (or organizational-level) job resources, e.g., supervisory coaching [4].

Possible limitations of the present study should be considered. First, the present study used list-wise deletion to determine the final sample for the analyses, comprising younger individuals and with higher education compared to the excluded sample. Because previous studies reported that individuals with lower education experienced greater job stress and lower job satisfaction [47], [48], these selection biases may underestimate the true association. Second, as mentioned earlier, information about employment contract was not obtained from each participant in the present study, which may have confounded the association of job demands with work engagement. Third, job demands, job resources, and work engagement were measured by self-report. Therefore, respondents might try to correlate their responses with their answers to previous questions to avoid cognitive dissonance. This common response bias may overestimate the true association. However, several studies have shown that these influences are not as strong as one could expect [49]. Fourth, although our sample was selected from a number of different industries and a wide variety of occupations, it comprised a higher percentage of employees from large-scale enterprises as well as permanent, non-manual, and male workers compared to general Japanese working population [50]. Therefore, the present findings may strongly reflect the features of these demographical groups, while they may underestimate or neglect the features of their counterparts (i.e., employees at medium- or small-scale enterprises, non-permanent, manual, and female workers). For this reason, generalization of the findings should be done with caution. Fifth, since we measured only workload and time pressure as a challenge and role ambiguity as a hindrance, future research should consider other kinds of challenges (e.g., cognitive demands) and hindrances (e.g., role conflict, interpersonal conflict, work-home interference) to provide more concrete evidence for the association of job demands with work engagement. Sixth, the present study did not measure personal resources (i.e., self-efficacy, OBSE, and optimism) which were reported to be associated with work engagement [51]. Further study considering job demands and job resources in conjunction with personal resources is promising, which may enrich theoretical and practical value of the JD-R model. Finally, the present study used a cross-sectional design while there are so many prospective studies tackling the same hypotheses in European countries [12]–[18]. However, the present study is the first to demonstrate that challenges and hindrances are differently associated with work engagement among Japanese working population. To address these limitations, the present study should be perceived as preliminary, and a prospective study is needed to assess more precise association of various job demands and job resources with work engagement using wide range of employees in small- to medium-scale enterprises in Japan.
